# Expanding the Scope of Microvascular Inflammation: Unveiling Its Presence Beyond Antibody-Mediated Rejection Into T-Cell Mediated Contexts

**DOI:** 10.3389/ti.2024.13464

**Published:** 2025-01-06

**Authors:** Hilal Varol, Anne Wagenmakers, Konrad Hoeft, Jasper Callemeyn, Roos Bodewes, Wichor Bramer, Andrew Stubbs, Rafael Kramann, Maarten Naesens, Marian C. Clahsen-Van Groningen

**Affiliations:** ^1^ Department of Pathology, Erasmus Medical Center Rotterdam, Rotterdam, Netherlands; ^2^ Erasmus MC Transplant Institute, University Medical Center Rotterdam, Rotterdam, Netherlands; ^3^ Division of Nephrology and Clinical Immunology, RWTH Aachen University Hospital, Aachen, Germany; ^4^ Department of Internal Medicine, Division of Nephrology and Transplantation, Erasmus University Medical Center Rotterdam, Rotterdam, Netherlands; ^5^ Division of Nephrology, University Hospitals Leuven, Leuven, Belgium; ^6^ Department of Microbiology, Immunology and Transplantation, Nephrology and Renal Transplantation Research Group, KU Leuven, Leuven, Belgium; ^7^ Medical Library, Erasmus MC, Erasmus University Medical Centre Rotterdam, Rotterdam, Netherlands; ^8^ Department of Pathology, Clinical Bioinformatics Unit, Erasmus MC, University Medical Center Rotterdam, Rotterdam, Netherlands

**Keywords:** kideny transplantation, banff classification, MVI, histology, TCMR

## Abstract

Microvascular inflammation (MVI) in kidney transplant biopsies is mainly associated with antibody-mediated rejection (AMR), sparking debate within the Banff Classification of Renal Allograft Pathology regarding its exclusivity. This study reviewed the literature on MVI in T cell-mediated rejection (TCMR) and analyzed MVI in our transplant population. We searched English publications in MEDLINE, Embase, Web of Science, Cochrane, and Google Scholar until June 2024, focusing on glomerulitis (g), peritubular capillaritis (ptc), or MVI in kidney transplant biopsies classified as TCMR. Additionally, we examined g, ptc, and MVI in 69 patients with AMR, TCMR, and no rejection. Our search yielded 541 citations, with 10 studies included, covering 810 TCMR and 156 AMR biopsies. The studies showed g, ptc, and MVI were present in TCMR but were less prevalent and severe than in AMR. In our cohort, AMR had significantly higher g, ptc, and MVI scores compared to aTCMR and ATN, however, aTCMR also displayed MVI. These findings confirm that MVI occurs in aTCMR and should not be exclusively linked to AMR. These findings highlight the need to further explore MVI’s significance in TCMR and investigate the inflammatory composition. This could refine the Banff Classification, improving Classification accuracy of kidney transplant pathology assessments.

## Graphical Abstract

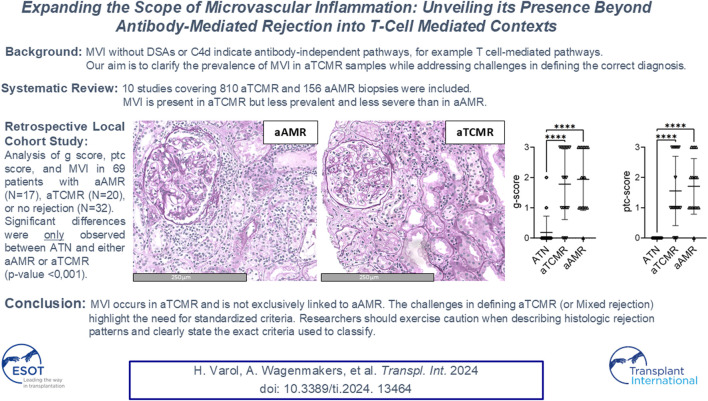

## Introduction

Since its inception in 1991, “the Banff Classification of Renal Allograft Pathology” provides diagnostic criteria for interpreting renal allograft pathology, evolving through biannual updates from experts [1]. It categorizes acute inflammatory lesions into specific clusters such as tubulitis (t), interstitial inflammation (i), glomerulitis (g) and peritubular capillaritis (ptc). The 2019 Banff update focused on histological evidence, primarily microvascular inflammation (MVI), and includes the presence of donor-specific antibodies (DSAs) and C4d deposition in peritubular capillaries as diagnostic criteria for antibody-mediated rejection (AMR). However, despite the comprehensiveness of the Banff Classification, it presents a challenge, as a considerable number of biopsies exhibit a high MVI-score ([g + ptc] ≥ 2) without detectable DSAs or C4d. The clinical implications of such findings are unclear [2]. The Banff Classification solely includes MVI in the category of AMR and does not provide a specific evaluation framework for MVI in other categories. Recently, there have been discussions about whether dichotomization of a complex histological image and thereby assigning MVI to AMR is valid. The 2022 Banff update addresses these complexities by identifying two new phenotypes of MVI and providing criteria for DSA- and C4d-negative cases, potentially involving various factors such as alloreactive T cell-mediated responses, non-HLA antibodies, primary NK cell activation through missing self, viral infection, other mechanisms of innate immune activation, and ischemia-reperfusion injury [3, 4]. In this report, we will conduct a systematic review investigating the literature’s perspective on MVI within the context of TCMR. Additionally, we will investigate the relationship between MVI and aTCMR and active AMR (aAMR) in our transplant population. We investigate the prevalence of MVI in cases classified as acute rejection as defined by the Banff Classification and to address the challenges in defining the correct diagnostic category.

## Materials and Methods

### Systematic Review

A systematic review of the literature was performed in accordance with Preferred Reporting Items for Systematic Reviews and Meta-Analyses (PRISMA) guidelines [5].

### Study Eligibility

#### Patients and Biopsies

With a focus on MVI within TCMR cases lacking an antibody-mediated component, our inclusion criteria comprised studies that examined kidney transplant biopsies with TCMR. Citations exclusively investigating AMR or Mixed Rejection without a clear description of the diagnostic process were excluded from consideration. Also, articles solely focusing on cases classified as suspicious (borderline) for acute TCMR were excluded due to the heterogeneous diagnostic grouping and the difference in Banff scoring of this category over time.

#### Index Test

Studies reporting the MVI score in TCMR transplant biopsies were eligible for inclusion. Studies only reporting g and/or ptc in TCMR allograft samples were also eligible for inclusion. Citations that excluded TCMR samples with g and/or ptc were excluded for this systematic review, as that the Banff Classification never explicitly mentions excluding the diagnosis of TCMR if MVI is present. We did not impose a minimum sample size (TCMR) in our selection as our hypothesis is that little is published on MVI in TCMR.

#### Comparators

Studies with lesion scores (specifically g, ptc or MVI) in TCMR samples compared to samples with other rejection patterns were considered for inclusion. Studies that mentioned lesion scores in different TCMR samples were also considered for inclusion. Studies that focused on chronic damage and not specifically on inflammation were excluded from further analysis.

#### Outcomes

Studies reporting individual lesion scores and/or MVI in the results section or Supplementary Material were included.

#### Study Design

English case-series, cross-sectional studies, cohort studies and controlled trials focusing on g, ptc or MVI in TCMR allograft biopsies in KTx were eligible for inclusion in the systematic review. We excluded manuscripts reporting non-original data.

### Information Sources and Search Strategy

We conducted a comprehensive literature search (Supplementary Material), regardless of language or publication status. An experienced information specialist (WB) developed database-specific search strategies for each of the following electronic databases (up to June 24, 2024): MEDLINE, EMBASE, Web of Science Core Collection, the Cochrane Central Register of Controlled Trials, and Google Scholar. The electronic search focused on MVI, allograft rejection, or failure. The electronic database searches were supplemented by manual scanning of the reference lists of relevant articles and reviews.

### Study Selection and Data Collection

The electronic database search yielded citations that we downloaded into Endnotes reference manager for screening. The selection process for eligible studies involved two stages: firstly, screening of titles and/or abstracts based on pre-established eligibility criteria, and secondly, conducting full-text evaluations of citations that were not excluded in the initial step by applying the same criteria. Relevant information from electronic database searches and potentially relevant full-text articles were screened independently by two investigators (AW, RB). Any disagreements were resolved through consensus and, if required, a third reviewer (HV) was involved.

### Data Items

After selecting relevant papers, two investigators (AW, HV) extracted the following data by reading the articles thoroughly: study design, sample size per rejection type (TCMR or AMR), sample size per biopsy type (indication or protocol) and Banff Classification used for diagnosis. Data on g, ptc and/or MVI were collected, as well as C4d and anti HLA-DSA status. Each article was evaluated on the method and details pertaining to the calculation of MVI. The quantitative representation of the g, ptc, MVI, C4d and non HLA-DSA status were assessed and evaluated whether they are comparable between the articles. Cases with BK nephropathy or (recurrence of) primary glomerulopathy were excluded from further analysis.

### Retrospective Cohort Study

#### Sample Collection

A total of 69 for-cause kidney transplant biopsies diagnosed as aAMR (n = 32), aTCMR (n = 20), and acute tubular necrosis (ATN) (n = 17), obtained between 2009 and 2019 previously included in a study investigating transcriptomics were retrieved from the archives of the ErasmusMC, Rotterdam, the Netherlands (MEC-2019-0307 [6]). All biopsies were re-evaluated according to the Banff 22 Update [2], focusing on Banff lesion scores g and ptc. Cases with aTCMR were excluded if they showed concurrent DSAs and/or C4d positivity. To support the robustness of our findings, we refer to previous transcriptional analysis of the samples as previously described [6]. These analyses found no distinct subgroups within the aTCMR group denoting an AMR transcription al profile, highlighting the uniformity of sample characteristics and reinforcing the integrity of our comparative assessments.

### Statistical Analysis

Statistical analysis was performed using SPSS software version 25 (IBM Corp., 2017). To assess whether there are significant differences in the Banff lesion scores for g, ptc and MVI between aAMR, aTCMR, and ATN, the Kruskal-Wallis test will be used. Post-hoc pairwise comparisons will be conducted using Dunn’s test with Bonferroni correction to identify specific group differences if the Kruskal-Wallis test yields a significant result. Statistical significance will be set at p < 0.05.

## Results

**FIGURE 1:**
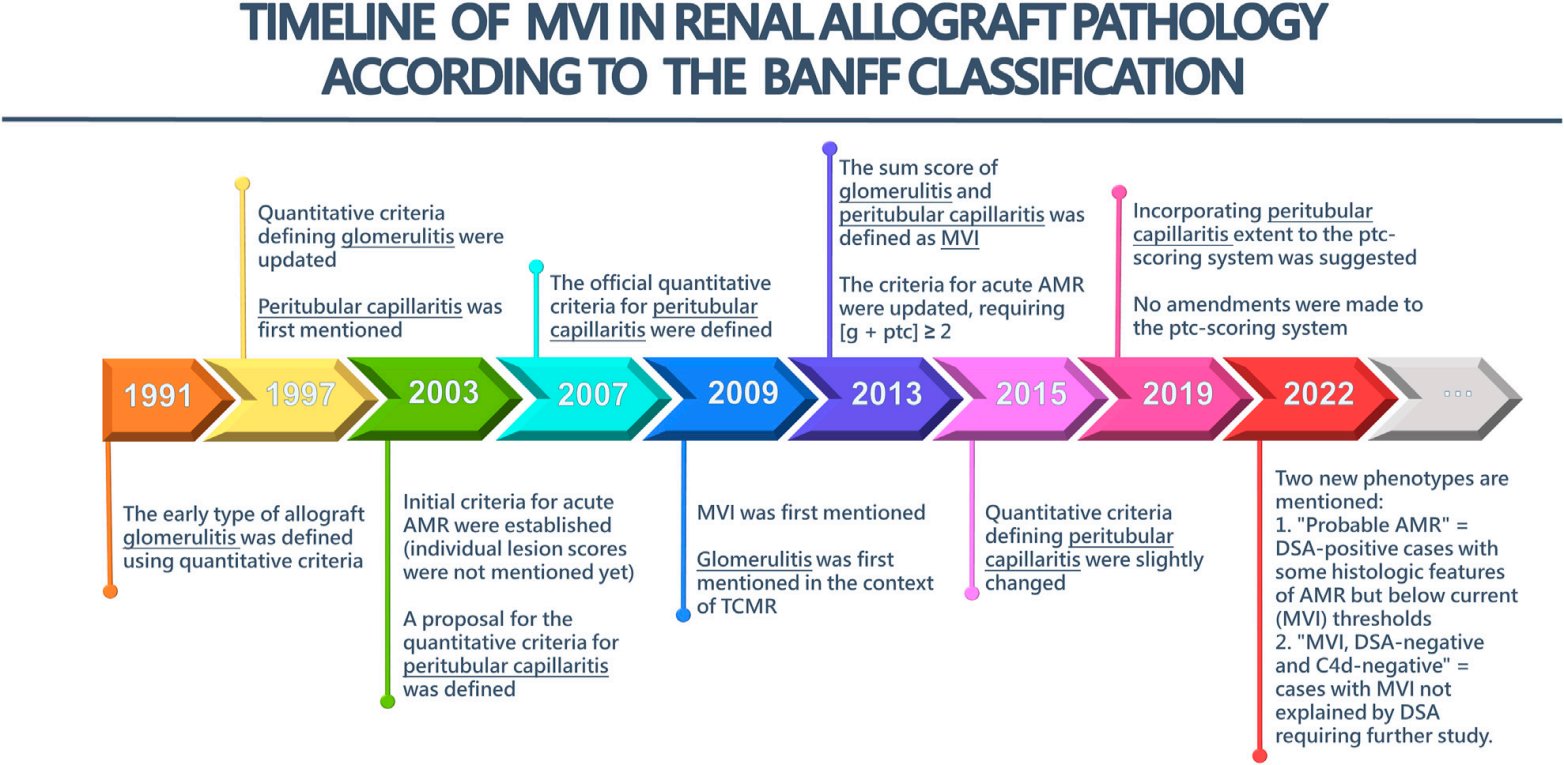
The timeline of microvascular inflammation (MVI) in Renal Allograft Pathology according to the Banff Classification. AMR, antibody-mediated rejection; TCMR, T cell-mediated rejection; g, glomerulitis; ptc, peritubular capillaritis.

The origin and timeline of MVI in renal allograft pathology according to the Banff Classification is depicted in Figure 1, with the most recent edition from 2022. Supplementary Table S1 provides a more detailed description of the key updates in the Banff Classification over the years, focusing on g-lesion score, ptc-lesion score, and MVI-score.

### Systematic Review

**FIGURE 2:**
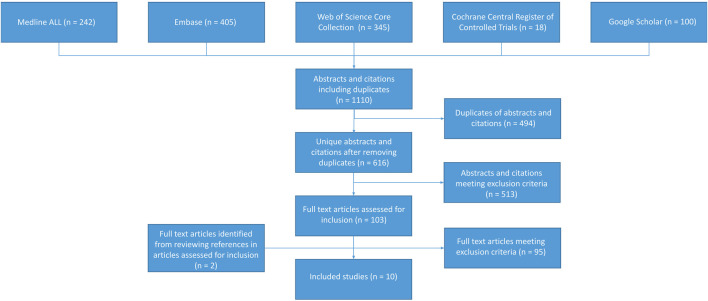
Summary of study inclusion and exclusion process.

Our search retrieved 616 citations after removing duplicates (Table 1). Of the 96 potentially relevant papers, eight studies met our criteria (Figure 2). After reviewing these eight articles, we found two additional studies meeting our criteria, which were not part of the initial 541 citations. Eight studies reported individual lesion scores (g and/or ptc) in renal transplant biopsies, and two studies reported MVI scores. Four studies reported C4d and anti-HLA DSA status. The excluded articles solely focused on mixed rejection or suspicious (borderline) for acute TCMR. Mixed rejection articles were omitted due to the presence of an AMR component, while articles on suspicious TCMR were excluded due to inconsistent definitions within this diagnostic group. A total of 2119 biopsies were included, of which 810 were renal allograft biopsies with TCMR. Among them, 1785 were indication biopsies, 577 were surveillance biopsies, and 46 were preimplantation biopsies. Additionally, the included articles covered a variety of rejection patterns besides TCMR. Furthermore, the included articles included samples with a broad range of different rejection patterns besides TCMR.

**TABLE 1:** Systematic review search strategy.

Database searched	Platform	Years of coverage	Records	Records after duplicates removed
Medline ALL	Ovid	1946 - June 2024	242	241
Embase	Embase.com	1971 - June 2024	405	188
Web of Science Core Collection^a^	Web of Knowledge	1975 - June 2024	345	113
Cochrane Central Register of Controlled Trials^b^	Wiley	1992 - June 2024	18	11
Additional Search Engines: Google Scholar^c^			100	63
Total			1,110	616

^a^ Science Citation Index Expanded (1975-present); Social Sciences Citation Index (1975-present); Arts and Humanities Citation Index (1975-present); Conference Proceedings Citation Index- Science (1990-present); Conference Proceedings Citation Index- Social Science & Humanities (1990-present); Emerging Sources Citation Index (2005-present).

^b^ Manually deleted abstracts from trial registries.

^c^ Google Scholar was searched via “Publish or Perish” to download the results in EndNote.

No other database limits were used than those specified in the search strategies.

### Clinical and Methodological Heterogeneity Among studies

Table 2 outlines the study characteristics. Methodological variations were noted across the included articles, particularly in their use of different editions of the Banff Classification. Despite this, there was consistency in the criteria for g and ptc lesion scores and MVI scores. However, the quantitative representation of the lesion scores varied. Comparing MVI scores between articles and different rejection patterns posed challenges due to the absence of a defined framework beyond AMR diagnostic criteria. According to the 2019 Banff Classification, AMR requires at least (g + ptc) ≥ 2. However, in the presence of acute TCMR, borderline infiltrate or infection, ptc ≥2 alone is insufficient, and g must be ≥1. Additionally, it was unclear whether AMR cases also included a TCMR component.

**TABLE 2:** Heterogeneity among studies in sample size and used Banff Classification.

Author, year	Setting	Design	Total biopsies –TCMR biopsies	Indication biopsies –Protocol biopsies	Banff classification used for diagnosis
Bouatou et al. [7]	Assistance Publique - Hôpitaux de Paris, Paris, France	Cohort study	256–256	256–0	2017
Jung et al. [8]	Kyungpook National University School of Medicine, Daegu, Korea	Cohort study	106–6	24–82	2013
Zhao et al. [9]	Beijing Friendship Hospital, Capital Medical University, Beijing, China	Cohort study	42–18	42–0	2013
Sis et al. [10]	University of Alberta, Edmonton, Alberta, Canada	Cohort study	329–44	329–0	2009
Lee et al. [11]	Kyung Hee University, Seoul, South Korea	Cohort study	203–46	169–34	2007
Park et al. [12]	Keimyung University Kidney Institute, Daegu, Korea	Cohort study	139–48	139–0	Unknown (biopsies from 2006 till 2018)
Gibson et al. [13].^a^	MS4 Health Sciences Centre, Winnipeg, Manitoba, Canada	Cohort study	688–76	181–461	2003
Gupta et al. [14]	Albert Einstein College of Medicine, Bronx, New York, USA	Cohort study	356–27	356–0	2013
Kozakowski et al. [15]	Medical University of Vienna, Vienna, Austria	Cohort study	1,322–224	1,322–0	2005, the composition of ptc was assessed in a different quantitative manner.^b^
Batal et al. [16]	University of Pittsburgh Medical Center, Pittsburgh, PA	Cohort study	111–65	111–0	2007

Abbreviations: (g): glomerulitis, (ptc): peritubular capillaritis, MVI: microvascular inflammation, TCMR: T cell-mediated rejection, AMR: antibody-mediated rejection.

^a^ An additional 46 preimplantation biopsies were included in this study.

^b^ The quantification of leukocytic composition of ptc was as follows: (i) predominantly mononuclear (>75% mononuclear cells), when mononuclear cells were at least three times as many as granulocytes; (ii) granulocytic dominated (>75% granulocytes), when granulocytes were at least three times as many as mononuclear cells; or (iii) mixed, if no dominant population was identified.

There is notable heterogeneity among the included articles regarding the definition and interpretation of different rejection patterns in relation to C4d and/or anti-HLA-DSA. Bouatou et al. excluded mixed rejection cases, yet 28 TCMR samples were DSA-positive [7]. In Park et al. 's article two TCMR cases were DSA-positive [12]. It’s unclear why they were not classified as mixed rejection. Sis et al. defined mixed rejection as TCMR plus AMR (C4d positive or negative), but did not provide the C4d and anti-HLA-DSA status for mixed rejection and TCMR cases [10]. Lastly, Zhao et al. selected 356 TCMR cases of which 197 were DSA-positive. Of the 159 DSA-negative TCMR cases a total of 131 cases were C4d positive [9].

### Glomerulitis in TCMR

Glomerulitis scoring ranges from 0 to 3, reflecting the percentage of glomeruli affected [17]. Among the included studies, six discussed g lesion scores (Table 3). Bouatou et al. found that 94.9% of acute TCMR cases had g0, and 5.1% had g1, with no cases with g2 or g3 [7]. In Zhao et al.’s study, borderline TCMR had a g lesion score of 0.10 ± 0.31 (mean ± SD), while acute TCMR had 0.25 ± 0.39 [9]. Sis et al. showed that 79% of TCMR samples displayed g0, while the remaining 21% exhibited a g > 0. In contrast, among the C4d-positive AMR samples, 40% had g0, while 60% showed g > 0 [10]. Lee et al. reported a median of 0 (0.1) for g in TCMR, while acute AMR had a median of g2 [2, 3]. [11] Park et al. found 91.7% of TCMR cases with g between 0% and 1% and 8.3% between g2–g3, while for AMR, 55.2% had g0–g1, and 44.8% had g2–g3 [12]. Lastly, Batal et al. reported that 37% of the TCMR samples had g0, 25% g1 score, 21% g2 score and 17% g3 [16]. Additionally, in Supplementary Table S3, the i score, t score, and v score are provided per article, where available, alongside the g score.

**TABLE 3:** Heterogeneity among studies in outcome variables in g, ptc and MVI in TCMR and AMR biopsies.

Author, year	Measurement	TCMR (n = 810)					AMR (n = 156)				
		g score	ptc score	MVI score (g + ptc)	C4d	Anti-HLA DSA	g score	ptc score	MVI score (g + ptc)	C4d	Anti-HLA DSA
Bouatou et al. [7] ^a^ ^,^ ^b^	N (%)	0: 243 (94.9) 1: 13 (5.1) 2: 0 (0) 3: 0 (0)	0: 149 (58.2) 1: 42 (16.4) 2: 44 (17.2) 3: 21 (8.2)	-	0: 256 (100) >0: 0 (0)	28 (11)	-	-	-	-	-
Jung et al. [8] ^c^ ^,^ ^d^	N (%)	-	-	0: 1 (9.1) ≥1: 5 (38.5)	-	-	-	-	0: 0 (0) ≥1: 5 (38.5)	2 (15.4)	-
Zhao et al. [9] ^a^ ^,^ ^e^	Mean (SD)	0.25 (0.39)	0.72 (0.89)	-	0 (0)	0 (0)	-	-	-	-	-
Sis et al. [10] ^f^	N (%)	0: 35 (79) >0: 9 (21)	0: 37 (84) >0: 7 (16)	0: 31 (71) >0: 13 (29)	-	In MVI>0: 0 (0)	0: 12 (40) >0: 18 (60)	0: 4 (13) >0: 26 (87)	0: 2 (7) >0: 28 (93)	30 (100)	In MVI>0: 28 (100)
							0: 20 (50) >0: 20 (50)	0: 14 (35) >0: 26 (65)	0: 11 (27) >0: 29 (73)	0 (0)	In MVI>0: 29 (100)
Lee et al. [11] ^b^ ^,^ ^c^	Median (IQR)	0 (0.1)	0 (0.0)	0 (0.1)	-	-	2 (2.3)	2 (2.3)	4 (3.5)	-	-
Park et al. [12]	N (%)	0-1: 44 (91.7) 2-3: 4 (8.3)	0-1: 42 (87.5) 2-3: 6 (12.5)	>1: 18 (37.5)	>0: 13 (27)	2 (11)	0-1: 32 (55.2) 2-3: 26 (44.8)	0-1: 23 (39.7) 2-3: 35 (60.3)	>1: 54 (93.1)	>0: 40 (69)	41 (68)
Gibson et al. [13]	N (%)	-	>0: 76 (68.4)	-	-	-	-	-	-	-	-
Gupta et al. [14] ^b^ ^,^ ^c^ ^,^ ^d^	N (%)	-	-	0: 8 (4) 1: 7 (10) ≥2: 12 (15)	-	-	-	-	0: 0 (0) 1: 2 (3) ≥2: 12 (15)	-	-
Kozakowski et al. [15]	N (%)	-	0: 137 (24.1) >0: 87 (48.1)	-	-	-	-	-	-	-	-
Batal et al. [16]	N (%)	0: 24 (37) 1: 16 (25) 2: 14 (21) 3: 11 (17)	-	-	-	-	0: 3 (30) 1: 3 (30) 2: 3 (30) 3: 1 (10)	-	-	-	-

Abbreviations: (g), glomerulitis; (ptc), peritubular capillaritis; MVI, microvascular inflammation; TCMR, T cell-mediated rejection; AMR, antibody-mediated rejection; SD, standard deviation; IQR, interquartile range.

^a^ This study focused on TCMR. Data on AMR was not included.

^b^ This study specifically included acute TCMR cases.

^c^ This study specifically included acute AMR cases.

^d^ This study focused on MVI in different diagnostic groups. The outcome (N) represents the number of samples of acute TCMR or acute AMR within each MVI group. The percentages are specific to MVI groups, not diagnosis categories. Diagnoses other than acute TCMR and acute AMR are not covered in this systematic review therefore the percentages do not sum up to 100%.

^e^ This study excluded all C4d and DSA positive TCMR cases, therefore the exact percentage of C4d and DSA positive TCMR cases is not clear.

^f^ Glomerulitis, peritubular capillaritis and MVI scores of TCMR and AMR samples are presented in this table. Data on C4d-positive and C4d-negative AMR cases are given separately.

### Peritubular Capillaritis in TCMR

Peritubular capillaritis can be scored 0 through 3, according to the presence and severity of ptc seen [17]. Of the studies that were included for this systematic review, seven studies investigated the ptc individual lesion score (Table 3). Bouatou et al. showed that 58.2% of the samples with acute TCMR had ptc0, whereas 16.4% had ptc1, 17.2% had ptc2 and 8.2% had ptc3 [7]. Zhao et al., showed that the borderline TCMR group had a ptc of 0.10 ± 0.31 (mean ± SD), whereas for acute TCMR group, it was 0.72 ± 0.89 (mean ± SD). These values were also compared to the control group as mentioned in the previous paragraph [9]. Sis et al. found that a majority of TCMR samples (84%) had a ptc0, while the remaining 16% had ptc>0. In contrast, 13% of the C4d-positive AMR samples had ptc0 while the majority (87%) had ptc>0 with a median (interquartile range) of 2 [2, 3]. [11] Park et al. showed that 87.5% of TCMR cases had a lesion score of ptc0–ptc1 and 12.5% had a lesion score for ptc2–ptc3. Whilst, 39.7% of the AMR samples had ptc0–ptc1 and a majority (60.3%) had ptc2–ptc3 [12]. Gibson et al. found that 68.4% of the TCMR samples had ptc, with a preponderance of ptc2. However, 68.6% of focal C4d-positive and 88.2% of diffuse C4d-positive samples had ptc. Diffuse C4d-positive samples comprised the majority of ptc3 in this cohort [13]. Lastly, Kozakowski et al. reported that 24.1% of samples classified as TCMR had ptc0 and 48.1% of samples had ptc>0 [15]. The other included studies did not report the distribution of the cases across ptc0, ptc1, ptc2, and ptc3 in the results section or in Supplementary Material. Additionally, in Supplementary Table S3, the i score, t score, and v score are provided per article, where available, alongside the ptc score.

### MVI in TCMR

Among the studies that were included for this systematic review, five studies investigated MVI (Table 3). In these studies, the MVI score was assessed by combining the Banff lesion scores g and ptc. Jung et al. reported that in cases with no MVI, there were no cases of acute AMR and only 9.1% had TCMR, while in cases with MVI≥1, the prevalence of both acute AMR and TCMR was 38.5%. It should be noted, however, that only six TCMR cases were analyzed in this study [8]. According to Sis et al., 71% of the TCMR cases did not show MVI, while the remaining 29% showed MVI>0. In contrast, among the C4d-positive AMR cases, 7% showed no MVI (MVI = 0) while the majority (93%) showed MVI>0 [10]. Lee et al. found a median (interquartile range) of 0 (0.1) for MVI in TCMR cases, while acute AMR cases had a median (interquartile range) of 4 [3, 5]. [11] Park et al. showed that 37.5% of the TCMR samples showed MVI≥1, compared to 93.1% of the AMR samples [12]. Lastly, Gupta et al. showed that in samples with no MVI, there were no cases of acute AMR and only 4% had acute TCMR, while in samples with MVI≥2, the prevalence of both acute AMR and acute TCMR was 15%. In cases with MVI = 1, 3% were acute AMR and 10% acute TCMR [14].

### Retrospective Local Cohort Study

**FIGURE 3:**
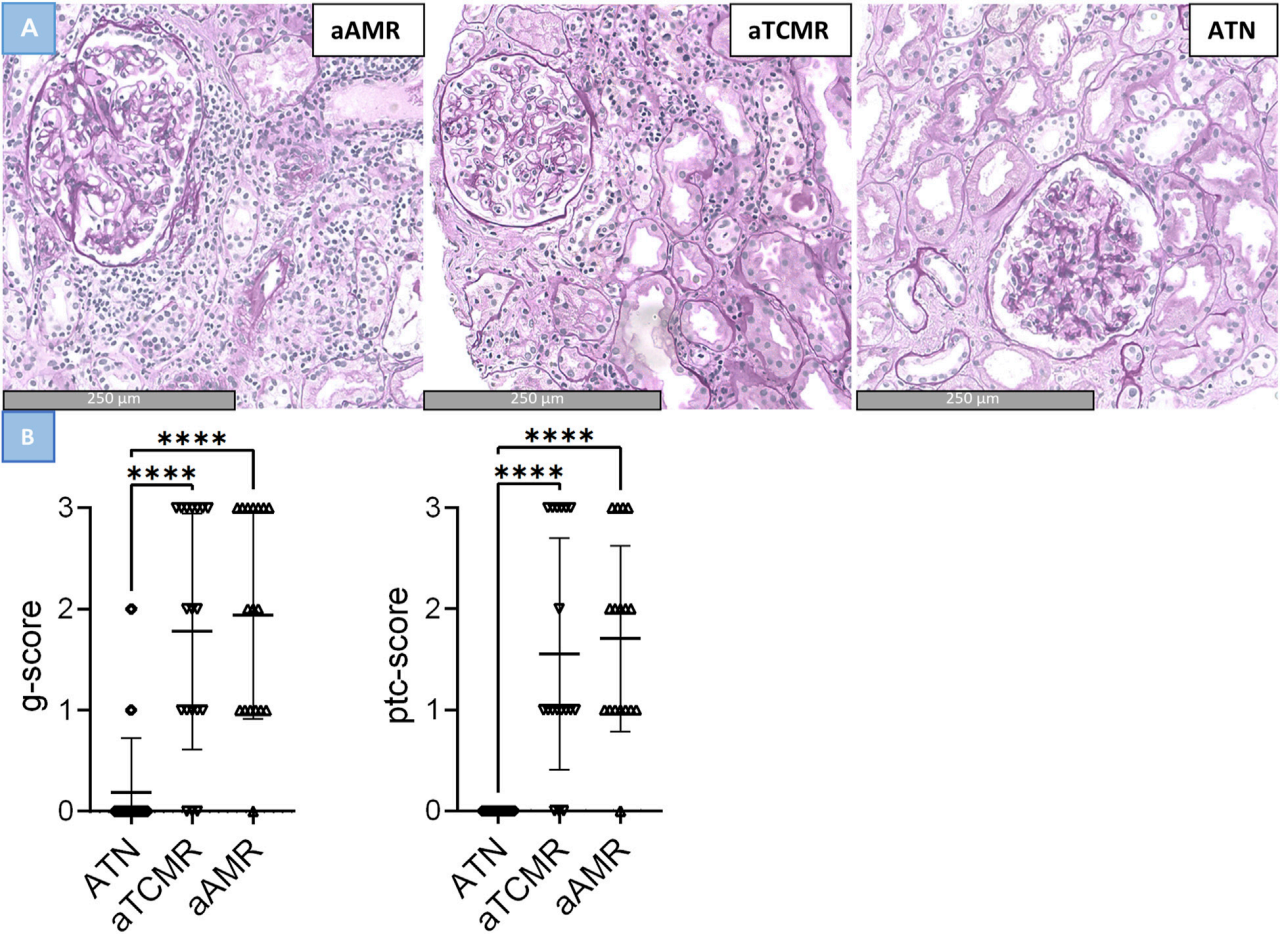
Differences in glomerulitis, peritubular capillaritis and MVI in active antibody–mediated rejection (aAMR), acute T cell-mediated rejection (aTCMR) and acute tubular necrosis (ATN). **(A)** Histomorphologic images of aAMR, aTCMR and ATN, respectively, showing microvascular inflammation (PAS+, magnification 40x). (B) Lession scores in ATN, aTCMR and aAMR. Kruskal-Wallis. ***P < 0.0001.

In our local study of 69 patients, cases with ATN had a g lesion score of 0.19 ± 0.54 (mean ± SD), a ptc lesion score of 0.00 ± 0.00 (mean ± SD), and an MVI score of 0.19 ± 0.54 (mean ± SD). Notably, two ATN cases demonstrated a g lesion score of g1 and two cases exhibited a g lesion score of g2. Cases classified as aTCMR, had a g lesion score of 1.60 ± 1.23 (mean ± SD), a ptc lesion score of 1.40 ± 1.19 (mean ± SD) and a MVI score of 3.00 ± 2.38 (mean ± SD). Interestingly, six cases of aTCMR had both a g lesion and ptc lesion score of 3 resulting in an MVI of 6. aAMR cases had a g lesion score of 2.00 ± 0.94 (mean ± SD), a ptc lesion score of 1.76 ± 0.83 (mean ± SD), and a MVI score of 3.76 ± 1.60 (mean ± SD). The g, ptc lesion and MVI scores per sample are depicted in Supplementary Table S2. Comparing the three groups, a significant difference was found between the g lesion score, the ptc lesion score and the MVI score (p-value < 0.001). However, upon conducting post-hoc pairwise comparisons, no significant difference was found between the aTCMR and aAMR groups in the g lesion score (p-value = 0.224), the ptc lesion score (p-value = 0.180) and MVI score (p-value = 0.224). Instead, significant differences were only observed between ATN and aTCMR or aAMR (p-value < 0.001), see Figure 3.

## Discussion

Our review reveals that the g and ptc score, along with the MVI score, are found in biopsies classified as aTCMR, albeit usually with lower frequency than in aAMR biopsies. Interestingly, our retrospective local cohort study also shows that MVI is present in aTCMR, additionally, we have found no differences between the degree of g, ptc and MVI between AMR and aTCMR.

Despite the elaboration in numerous studies, the correlation between scores for g, ptc, and MVI in TCMR or other renal transplant pathology remains unaddressed in the Banff Classification. Batal et al. showed that glomerulitis, commonly associated with AMR, was also detected in DSA-/C4d- TCMR (47%) and DSA/C4d-borderline samples (62%) [16]. Sis et al. showed that while glomerulitis occurs more frequently in AMR biopsies compared to TCMR or glomerulonephritis biopsies, its severity remains consistent across different types of renal allograft pathology, suggesting that glomerulitis is an ambiguous lesion [10]. Furthermore, Gupta et al. found that MVI is not specific for AMR and can also be seen in ATN, glomerulonephritis, and TCMR. In 19% of the for-cause transplant biopsies with MVI ≥2, there were no other histologic signs of rejection [14].

Interestingly, in our retrospective analysis, none of the cases with ATN had a ptc score greater than 0 (at least one leukocyte in <10% of cortical PTCs and/or maximum number of leukocytes <3), indicating that these cases either displayed no peritubular capillary inflammation or that any observed inflammation fell below the diagnostic threshold. This could be due to the highly stringent selection of the cases included. In addition to AMR and TCMR, it is important to recognize that some cases of ATN may also exhibit (ptc) or a lower score of MVI. In instances of ATN associated with MVI, it is crucial to carefully consider the implications and potential consequences of MVI. By closely monitoring these patients, we can gain valuable insights into their prognosis, as they might be at increased risk for developing rejection in some cases.

Recent research has expanded our understanding of MVI in organ transplantation, revealing multiple underlying causes beyond antibody-mediated mechanisms. Studies have shown significant NK cell presence in AMR cases with MVI, alongside specific AMR-related NK cell transcripts, including *SH2D1B*, *GNLY*, *FGFBP2*, and *CD160* [18–20]. Lamarthee et al. suggest that NK cells primarily interact with transplants through FCRL3 induction, triggering antibody-dependent cellular cytotoxicity [21]. Additionally, NK cells can recognize missing self by sensing the absence of HLA-I molecules through Killer Ig-Like Receptors, contributing to MVI [22–25]. Understanding these pathways holds therapeutic potential. Koening et al. showed that chronic vascular rejection depends on the mTORC1 pathway, with mTOR inhibitors showing promise in preventing development of histological lesions [25, 26]. Senev et al. suggested that antibody-independent NK cell activation mediated by the missing self is a mechanism through which HLA mismatch in the allorecognition pathway can lead to MVI. Interestingly, one-third of cases with this phenotype also showed concurrent TCMR, indicating possible primary T cell involvement in antibody- and complement-independent processes [27]. This relation between T cell activation and MVI has been demonstrated in preclinical research. T cells possess the ability to recognize all antigens, including HLA (class 2) antigens on endothelial cells, potentially leading to direct targeting of endothelial cells in transplanted tissue, causing microvascular injury and inflammation [28–30].

Understanding the diverse causes of MVI and their complex pathways is crucial for grasping their clinical effects on graft outcomes. In DSA+ AMR cases, MVI correlates with poor graft survival rates [31], especially when it progresses to chronic graft injury (cg), observed in both DSA-positive and DSA-negative cases [32]. Parajuli et al. discovered that DSA- AMR patients with MVI had similar poor graft survival rates as DSA+ AMR patients with MVI, but did not mention patients who had MVI alongside TCMR [24]. Our group has recently shown that cases showing transplant glomerulopathy lacking DSA and no C4d have superior transplant function to those cases classified as caAMR (ref Varol et al KI). Bouatou et al. identified factors independently associated with transplant loss in TCMR patients during the 3-month post-treatment assessment, which included GFR, proteinuria, i-IF/TA, anti-HLA DSA, and ptc score (HR, 2.27; 95% CI, 1.13-4.55; P = .022). However, the study did not differentiate between cases with positive and negative DSA status [7]. So, while both antibody-independent and antibody-dependent mechanisms can contribute to MVI, their relative impact remains uncertain. To address this gap, it is important to understand distinct infiltrating cell types and underlying pathophysiological mechanisms. Immunophenotyping and transcriptomics could aid in elucidating the differences in MVI between TCMR and AMR. Such insights hold considerable potential for diagnostic classification and enhancing the precision of treatment decisions.

The findings of our systematic review come with limitations. Initially, we included eight articles and found two more by reviewing their references. However, these additional articles were not part of our initial search, revealing a limitation in our search strategy. Despite our specific and thorough search, we acknowledge that we may have missed some studies addressing MVI in TCMR. In particular, studies published before MVI and peritubular capillaritis became an area of interest and discussion within the context of TCMR, their MeSH terms might not have been used widely. Limitation arises from the relatively small number of available articles eligible for reliable data extraction and subsequent analysis. Including articles that only mention the “g” or “ptc” score without providing the complete MVI score presents a limitation. Without this comprehensive MVI score, it becomes difficult to thoroughly evaluate the influence of these variables. Nonetheless, these articles still provide valuable insights into aspects related to MVI. If these papers were excluded, it would reduce the number of eligible articles, potentially affecting the overall comprehensiveness of the study. Furthermore, most of the included manuscripts are observational and retrospective, inherently introducing a layer of selection bias. In order to maintain the integrity of this study and prevent another layer of selection bias, we excluded articles that excluded TCMR biopsies featuring g, ptc, or MVI, as the Banff Classification previously did not explicitly mention including the diagnosis of TCMR if MVI is present. Consequently, our pool of eligible articles became even more limited, with insufficient sample sizes for statistical pooling methods.

Another important limitation arises from the ambiguity in defining and interpreting various rejection patterns. In all studies included, in addition to the MVI score typically being represented as mean, median, or percentage values and without access to individual sample data, there is no information on individual DSA status or C4d staining. We therefore cannot be 100% sure that those cases that the authors diagnosed as “pure” TCMR are indeed so. However, as they did follow the Banff classification, we do assume that they did take the factors of DSA and C4d into account when diagnosing the cases of TCMR. Additionally, there was heterogeneity among the included articles in lesion interpretation methods and Banff Classification versions used. While modifications and updates to the Banff classification are necessary, they make comparing studies over time challenging. Therefore, inconsistencies in TCMR and mixed rejection interpretations, together with varying diagnostic criteria descriptions, make it unclear whether differences in TCMR cases with g, ptc, or MVI among studies stem from some studies categorizing TCMR with MVI as mixed rejection, while others classify this as TCMR. This diversity in terminology and methodologies posed challenges in establishing direct comparisons between the results. Additionally, some of the included articles mentioned samples classified as borderline TCMR or mixed rejection alongside TCMR cases. We chose not to incorporate data of these samples in our analysis due to insufficient descriptions regarding the precise definitions of these diagnoses. It is for example unclear how pathologists would diagnose TCMR with MVI; some might classify it as mixed rejection whilst others might consider the MVI as part of TCMR and label it as “pure” TCMR. In mixed rejection, it is not possible to attribute the presence of MVI to either AMR or TCMR or maybe even both, as little is known about the inflammatory cells present in both Banff categories. Finally, it is known that TCMR is a risk factor for AMR [33], which could therefore possibly explain the presence of MVI in TCMR. This decision was made to mitigate another potential factor for ambiguity in interpreting the results. Nevertheless, it is worth noting that excluding this information might have limited the findings of our study.

Future research efforts should focus on elucidating the role of MVI in aTCMR, identifying the main contributing inflammatory cells, and exploring the molecular profile of MVI within this context. Incorporating advanced immunohistochemistry and immunofluorescent staining techniques may provide deeper insights into the specific immune cell populations involved. By addressing these challenges and uncertainties, we can enhance our understanding of transplant pathology and ultimately improve patient outcomes. This comprehensive approach will help clarify the inflammatory composition and facilitate the identification of potential therapeutic targets.

To summarize, our systematic review and retrospective cohort study shows that g, ptc, and MVI can be present in for-cause kidney transplant biopsies diagnosed with aTCMR. It is crucial to underscore that in a clinical setting a more detailed clinicopathological correlation is necessary for an accurate diagnosis. Our analysis also revealed challenges related to the definition and classification of aTCMR and mixed rejection across various articles. These challenges underscore the importance of standardized and clear criteria in defining rejection patterns, especially given the biannual updates to the Banff Classification. Moving forward, it is imperative for researchers to exercise caution when including and describing different rejection patterns, ensuring a thorough description of the exact criteria used for their included samples.

## Data Availability

The raw data supporting the conclusions of this article will be made available by the authors, without undue reservation.

## Abbreviations

AMR, antibody-mediated rejection; aAMR, active AMR; DSA, donor-specific antibodies; DSAST, DSA selective transcripts; ENDAT, endothelial-associated transcripts; g, glomerulitis; IFNγ, interferon-γ; MHC, major histocompatibility complex; MVI, microvascular inflammation; ptc, peritubular capillaritis; TCMR, T cell-mediated rejection; aTCMR, acute TCMR.
