# Supplementary material for: Expanding the Scope of Microvascular Inflammation: Unveiling Its Presence Beyond Antibody-Mediated Rejection Into T-Cell Mediated Contexts
Source: Transpl Int. 2025 Jan 6;37:13464. doi: 10.3389/ti.2024.13464 (PMC11742949; doi:10.3389/ti.2024.13464)
Supplement: Supplementary file 1 [file DataSheet1.docx]

The search strings per database were as follows,

Medline ALL Ovid

(Microvessels / OR (microvascul* OR microvessel* OR microcircul* OR micro-vascul* OR micro-vessel* OR micro-circul* OR capillar* OR glomerulitis* OR ((glomerulonephrit* OR glomerulopath*) ADJ3 (graft* OR transplant* OR allograft* OR allotransplant*))).ab,ti.) AND (exp Host vs Graft Reaction/ OR Primary Graft Dysfunction / OR Delayed Graft Function / OR (reject* OR ((glomerulonephrit* OR glomerulopath* OR nephropath* OR dysfunction* OR failure* OR delay* OR surviv* OR loss*) ADJ3 (graft* OR transplant* OR allograft* OR allotransplant*))).ab,ti.) AND (Kidney Transplantation / OR (((kidney OR renal) ADJ3 (transplant* OR allotransplant* OR reject* OR graft* OR allograft*))).ab,ti.) AND (exp T-Lymphocytes / OR (T-lymphocyte* OR t-cell* OR TCMR OR T-regulator* OR treg).ab,ti.) NOT (exp animals/ NOT humans/) AND english.la.

Embase.com

(microvasculature/exp OR glomerulitis/de OR glomerulopathy/de OR capillaritis/de OR (microvascul* OR microvessel* OR microcircul* OR micro-vascul* OR micro-vessel* OR micro-circul* OR capillar* OR glomerulitis* OR ((glomerulonephrit* OR glomerulopath*) NEAR/3 (graft* OR transplant* OR allograft* OR allotransplant*))):ab,ti) AND ('graft rejection'/de OR 'kidney allograft rejection'/de OR 'kidney graft rejection'/de OR 'acute graft rejection'/de OR 'acute vascular rejection'/de OR 'chronic graft rejection'/de OR 'hyperacute graft rejection'/de OR 'chronic allograft nephropathy'/de OR 'graft dysfunction'/exp OR 'graft survival'/de OR (reject* OR ((glomerulonephrit* OR glomerulopath* OR nephropath* OR dysfunction* OR failure* OR delay* OR surviv* OR loss*) NEAR/3 (graft* OR transplant* OR allograft* OR allotransplant*))):Ab,ti) AND ('kidney transplantation'/exp OR 'kidney allograft rejection'/de OR 'kidney graft rejection'/de OR 'renal graft dysfunction'/de OR 'chronic allograft nephropathy'/de OR (((kidney OR renal) NEAR/3 (transplant* OR allotransplant* OR reject* OR graft* OR allograft*))):Ab,ti) AND ('T lymphocyte'/exp OR (t-lymphocyte* OR t-cell* OR TCMR OR T-regulator* OR treg):ab,ti) NOT ([animals]/lim NOT [humans]/lim) NOT [conference abstract]/lim AND [english]/lim

Web of science

TS=(((microvascul* OR microvessel* OR microcircul* OR micro-vascul* OR micro-vessel* OR micro-circul* OR capillar* OR glomerulitis* OR ((glomerulonephrit* OR glomerulopath*) NEAR/2 (graft* OR transplant* OR allograft* OR allotransplant*)))) AND ((reject* OR ((glomerulonephrit* OR glomerulopath* OR nephropath* OR dysfunction* OR failure* OR delay* OR surviv* OR loss*) NEAR/2 (graft* OR transplant* OR allograft* OR allotransplant*)))) AND ((((kidney OR renal) NEAR/2 (transplant* OR allotransplant* OR reject* OR graft* OR allograft*)))) AND ((T-lymphocyte* OR t-cell* OR TCMR OR T-regulator* OR treg))) NOT DT=(Meeting Abstract OR Meeting Summary) AND LA=(english)

Cochrane

((microvascul* OR microvessel* OR microcircul* OR micro-vascul* OR micro-vessel* OR micro-circul* OR capillar* OR glomerulitis* OR ((glomerulonephrit* OR glomerulopath*) NEAR/3 (graft* OR transplant* OR allograft* OR allotransplant*))):ab,ti) AND ((reject* OR ((glomerulonephrit* OR glomerulopath* OR nephropath* OR dysfunction* OR failure* OR delay* OR surviv* OR loss*) NEAR/3 (graft* OR transplant* OR allograft* OR allotransplant*))):Ab,ti) AND ((((kidney OR renal) NEAR/3 (transplant* OR allotransplant* OR reject* OR graft* OR allograft*))):Ab,ti) AND ((T-lymphocyte* OR t-cell* OR TCMR OR T-regulator* OR treg):ab,ti)

Google scholar

microvasculature|microvessels|capillary|glomerulitis|" graft|transplant|allograft|allotransplant glomerulonephritis|glomerulopathy'' rejection "kidney|renal transplantion|allotransplantion|graft|allograft" "T lymphocyte|cell|regulator|cells|lymphocytes''

**Table S1.** Key updates in Banff Classification over the year regarding MVI

| Year | Key Updates and Points of Interest |
| --- | --- |
| 1991 | - First Banff meeting establishing international standards for diagnosing renal allograft rejection.  - Introduces early allograft glomerulitis criteria: g0 (no glomerulitis), g1 (minority of glomeruli), g2 (25-75% of glomeruli), g3 (mostly global in nearly all glomeruli).  - Uncertainty on the significance of glomerulitis in rejection. |
| 1997 | - Refines glomerulitis criteria: g0 (none), g1 (<25%), g2 (25-75%), g3 (>75% glomeruli affected).  - Links endothelial damage, vasculitis, thromboses, and leukocytes in capillaries to an antibody-mediated factor.  - First mention of ptc involvement. |
| 2003 | - Establishes initial acute AMR criteria: antibody action, circulating antibodies to donor HLA, and morphological signs of injury (e.g., immune cell infiltration in peritubular capillaries and/or glomeruli).  - Monocyte/macrophage infiltration in glomeruli and peritubular capillaries is considered specific to AMR. |
| 2005 | - Introduces quantitative ptc criteria, with updates in 2007 and 2015. |
| 2011 | - Introduces the term microvascular inflammation (MVI).  - Identifies chronic microvascular remodeling in presensitized patients with subclinical microcirculation inflammation, even in C4d-negative cases with DSA.  - Notes g can occur in TCMR. These patients respond well to steroid treatment. |
| 2013 | - Defines the sum score of g and ptc as MVI.  - Updates criteria for diagnosing acute AMR: requires at least moderate MVI ([g + ptc] ≥ 2).  - Notes lack of specific MVI evaluation framework beyond AMR criteria. |
| 2019 | - Highlights challenge of biopsies showing [g + ptc] ≥ 2 without detectable anti-HLA DSAs or C4d, with unclear clinical implications.  - In these cases recommends considering differential diagnoses like T cell–mediated endothelial injury, membranoproliferative glomerulonephritis, and thrombotic microangiopathy. |
| 2022 | - Identifies two new MVI phenotypes for C4d- and/or DSA-negative cases:   - Categorizes cases with MVI but without DSA and C4d as "MVI, DSA-negative and C4d-negative." - Categorizes cases with MVI below the threshold, without C4d but with DSA, as “probable AMR.”   - Specifies that a g lesion score of 1 or higher is required to diagnose AMR in the presence of acute TCMR.  - Emphasizes need for further research on cases with MVI without DSA or C4d. |

**Table S2.** Overview of the lesion scores and MVI score of included cases

| Case | Diagnosis | g | ptc | MVI |
| --- | --- | --- | --- | --- |
| 1 | ATN | 0 | 0 | 0 |
| 2 | ATN | 0 | 0 | 0 |
| 3 | ATN | 1 | 0 | 1 |
| 4 | ATN | 0 | 0 | 0 |
| 5 | ATN | 2 | 0 | 2 |
| 6 | ATN | 0 | 0 | 0 |
| 7 | ATN | 0 | 0 | 0 |
| 8 | ATN | 0 | 0 | 0 |
| 9 | ATN | 0 | 0 | 0 |
| 10 | ATN | 0 | 0 | 0 |
| 11 | ATN | 0 | 0 | 0 |
| 12 | ATN | 0 | 0 | 0 |
| 13 | ATN | 0 | 0 | 0 |
| 14 | ATN | 0 | 0 | 0 |
| 15 | ATN | 0 | 0 | 0 |
| 16 | ATN | 0 | 0 | 0 |
| 17 | ATN | 0 | 0 | 0 |
| 18 | ATN | 0 | 0 | 0 |
| 19 | ATN | 0 | 0 | 0 |
| 20 | ATN | 0 | 0 | 0 |
| 21 | ATN | 0 | 0 | 0 |
| 22 | ATN | 0 | 0 | 0 |
| 23 | ATN | 0 | 0 | 0 |
| 24 | ATN | 1 | 0 | 1 |
| 25 | ATN | 0 | 0 | 0 |
| 26 | ATN | 0 | 0 | 0 |
| 27 | ATN | 0 | 0 | 0 |
| 28 | ATN | 2 | 0 | 2 |
| 29 | ATN | 0 | 0 | 0 |
| 30 | ATN | 0 | 0 | 0 |
| 31 | ATN | 0 | 0 | 0 |
| 32 | ATN | 0 | 0 | 0 |
| 33 | aTCMR2A | 0 | 0 | 0 |
| 34 | aTCMR2A | 3 | 3 | 6 |
| 35 | aTCMR2A | 0 | 0 | 0 |
| 36 | aTCMR2A | 2 | 1 | 3 |
| 37 | aTCMR2A | 3 | 3 | 6 |
| 38 | aTCMR2A | 1 | 1 | 2 |
| 39 | aTCMR2B | 1 | 1 | 2 |
| 40 | aTCMR2A | 2 | 1 | 3 |
| 41 | aTCMR1A | 1 | 1 | 2 |
| 42 | aTCMR2A | 3 | 3 | 6 |
| 43 | aTCMR2A | 3 | 3 | 6 |
| 44 | aTCMR2A | 2 | 1 | 3 |
| 45 | aTCMR2A | 3 | 3 | 6 |
| 46 | aTCMR2A | 3 | 3 | 6 |
| 47 | aTCMR2A | 1 | 1 | 2 |
| 48 | aTCMR2A | 0 | 0 | 0 |
| 49 | aTCMR1B | 0 | 0 | 0 |
| 50 | aTCMR1A | 0 | 0 | 0 |
| 51 | aTCMR2B | 3 | 2 | 5 |
| 52 | aTCMR2A | 1 | 1 | 2 |
| 53 | aAMR | 3 | 3 | 6 |
| 54 | aAMR | 1 | 1 | 2 |
| 55 | aAMR | 1 | 2 | 3 |
| 56 | aAMR | 1 | 1 | 2 |
| 57 | aAMR | 3 | 3 | 6 |
| 58 | aAMR | 3 | 2 | 5 |
| 59 | aAMR | 1 | 1 | 2 |
| 60 | aAMR | 2 | 3 | 5 |
| 61 | aAMR | 3 | 1 | 4 |
| 62 | aAMR | 2 | 2 | 4 |
| 63 | aAMR | 3 | 2 | 5 |
| 64 | aAMR | 2 | 1 | 3 |
| 65 | aAMR | 3 | 2 | 5 |
| 66 | aAMR | 3 | 3 | 6 |
| 67 | aAMR | 1 | 1 | 2 |
| 68 | aAMR | 1 | 1 | 2 |
| 69 | aAMR | 1 | 1 | 2 |
